# Supplementary material for: Effect of Small Extracellular Vesicles Produced by Mesenchymal Stem Cells on 5xFAD Mice Hippocampal Cultures
Source: Int J Mol Sci. 2025 Apr 24;26(9):4026. doi: 10.3390/ijms26094026 (PMC12071690; doi:10.3390/ijms26094026)
Supplement: Supplementary file 1 [file ijms-26-04026-s001.zip › ijms-3506749-supplementary.pdf]

Effect of small extracellular vesicles produced by mesenchymal stem cells on 5xFAD mice hippocampal cultures

Zhdanova Daria Y., Bobkova Natalia V., Chaplygina Alina V., Svirshchevskaya Elena V., Poltavtseva Rimma A., Vodennikova Anastasia A., Chernyshev Vasiliy S., Sukhikh Gennadiy T.

Methods

MSC characterization (surface marker assessment)

The MSCs were obtained from Wharton's jelly of the umbilical cord by employees of the Federal Institution, the Academician V.I. Kulakov Research Center for Obstetrics, Gynecology and Perinatology of the Ministry of Health of the Russian Federation who have a special permit for this type of work. The primary culture of MSCs was isolated from Wharton's jelly of the umbilical cord obtained after cesarean section from different (at least five) healthy examined women in labor. The material was collected with the written informed consent of the donors. The tissue samples were mechanically crushed and placed in a 0.1% solution of type I collagenase (Gibco, USA) for 60 min at 37°C. After incubation, the suspension was precipitated by centrifugation for 3 min at 200×g. The pellet was resuspended in DMEM (Gibco, USA)-F12 (PanEco, Russia) (1:1) with the addition of 10% fetal bovine serum (Gibco, USA) and 1% Penicillin-Streptomycin-Glutamine (Gibco, USA) and placed in 25 cm^2 culture flasks (Corning). Cells were cultured at 37°C and 5% CO_2. Cells that reached 80% confluency were detached with 0.05% trypsin (PanEco, Russia).

The phenotype of the isolated MSCs was characterized by flow cytometry using specific markers. Fluorescence intensity was analyzed using a FACSCalibur flow cytometer and BD CellQuest Pro software (BD Biosciences, USA). The cell population was isolated by forward (FSC) and side (SSC) light scattering parameters. The expression of markers such as CD90, CD105, CD73, CD19, and HLA-DR was assessed. The expression level of the studied marker was assessed by the fluorescence intensity histogram. Immunocytochemical staining was performed using monoclonal antibodies conjugated with phycoerythrin. For analysis, the cells were removed from the surface of the flask with a trypsin solution and washed twice with staining buffer (PBS, 1.0% fetal bovine serum, and 0.1% sodium azide). 15 μl of labeled antibodies to one of the studied surface markers were added to the cells, bringing the volume of the cell suspension to 100 μl. The corresponding isotype antibodies were added to the samples serving as a negative control. The cell suspension was incubated at +4°C for 1 hour. After incubation, the cells were washed 2 times in 1 ml of staining buffer and fixed in 0.5 ml of 2% paraformaldehyde solution. The resulting cell suspension was filtered through a filter with a pore diameter of 30 μm to exclude cell aggregates. Fluorescence intensity was analyzed using a FACSAria flow

cytofluorimeter-sorter (Becton Dickinson Co.). In further experiments, only MSCs that had undergone no more than 6 passages were used.

Results

As a result of the cytometric analysis, it was shown that the obtained culture of MSCs isolated from Wharton's jelly of human umbilical cord expressed markers CD73, CD90, CD105, recommended by the International Society for Cellular Therapy for identification of multipotent mesenchymal stromal cells (MMSCs), as well as the CD44 marker. Immunocytochemical staining of MSCs using specific monoclonal antibodies conjugated with fluorescein isothiocyanate revealed positive immunoreactivity of the isolated cells to CD90, CD105, CD73, CD44 and CD13. The obtained data allowed us to conclude that the isolated cell preparation consists of mesenchymal stem cells and does not contain admixtures of hematopoietic stem cells.

Table S1. Concentration of humoral factors (pg/mL) in MSC supernatants (MSC SN), flow through after sEV purification and in sEVs.

		Flow through		MSC SN		sEVs	
	Number of samples	6		12		6	
N		Average	SEM	Average	SEM	Average	SEM
1	EGF	7	0.8	11	0.3	14	7
2	FGF-2	49	3.8	62	5.8	60	35
3	Eotaxin	14	3.3	40	7.5	19	9
4	G-CSF	79	8.8	161	31	48	27
5	Flt-3L	13	1.3	20	1.4	19	9
6	GM-CSF	11	5.4	20	4.9	2	2
7	Fractalkine	140	29.6	287	41.9	67	39
8	IFNa2	18	4.2	35	5.5	13	6
9	IFNg	3	0.8	4	0.6	18	12
10	GRO	2045	111	3466	788	84	56
11	IL-10	5	1.3	9	0.9	5	3
12	MCP-3	112	89	16	3.8	20	11
13	IL-12P40	14	2.1	20	2.6	2	1
14	MDC	38	5.8	43	3.8	36	17
15	PDGF-AA	7	2.9	14	3.2	9	7
16	PDGF-AB/BB	16	2.9	18	2.6	15	9
17	sCD40L	21	4.6	24	2.6	1	0
18	IL-1RA	13	2.5	18	1.4	20	11
19	IL-1a	11	6.3	12	5.2	6	4
20	IL-4	82	37.9	167	37	31	16
21	IL-6	2791	845	3901	205	33	38
22	IL-7	38	9.6	53	2.3	10	5
23	IL-8	1737	729	2985	326	39	18
24	IP-10	59	15.8	89	4.0	28	14
25	MCP-1	4108	1054	5283	87	106	132
26	MIP-1a	6	0.8	9	0.9	66	20

27	MIP-1b	6	0.8	9	1.2	14	6
28	RANTES	12	2.1	22	4.9	14	7
29	TNFa	1	0.0	1	0.0	3	3
30	VEGF	57	4.6	80	10.7	125	41
	Pearson's coefficient, *r*	**0.992* (p<0.001)**				**0.504 (p<0.005)**	

* Correlation of data with MSC supernatant.

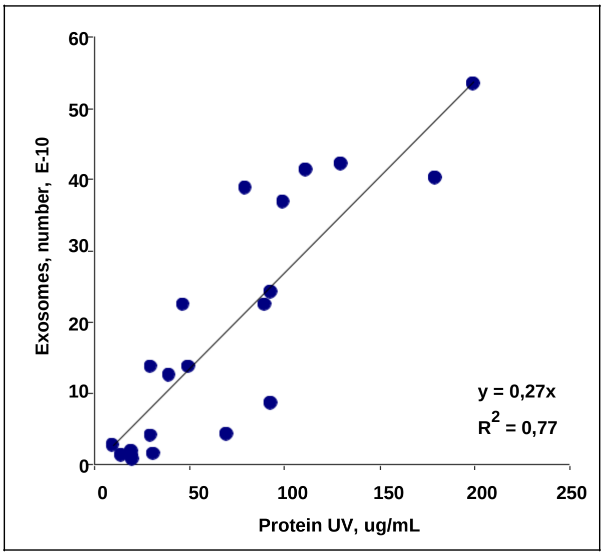

Figure S1. Extrapolation of sEV numbers measured by NTA (Y axis) and protein concentration measured by BCA or Nanodrop (X axis). Regression performed by using a linear function.

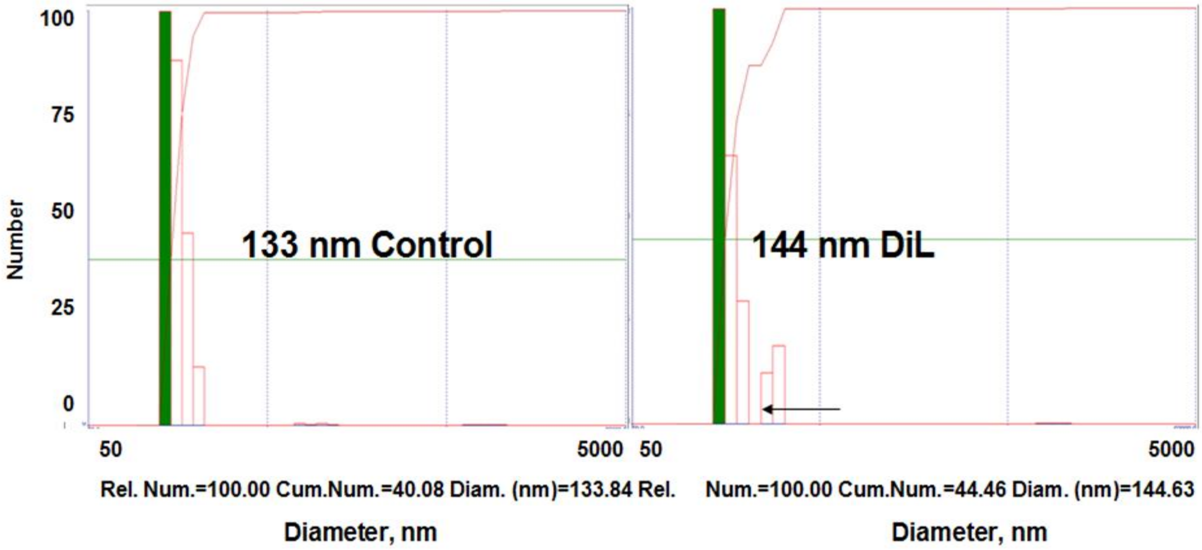

Figure S2. DLS of sEV purified from MSC before and after staining with DiL. sEV were kept frozen, then thawed, stained, and measured. Arrow shows aggregation of the sEVs after DiL staining.
